# Supplementary material for: Use of electronic health records to characterize patients with uncontrolled hypertension in two large health system networks
Source: BMC Cardiovasc Disord. 2024 Sep 18;24:497. doi: 10.1186/s12872-024-04161-x (PMC11409735; doi:10.1186/s12872-024-04161-x)
Supplement: Supplementary file 1 — Supplementary Material 1. [file 12872_2024_4161_MOESM1_ESM.docx]

**SUPPLEMENTAL MATERIAL**

**Use of Electronic Health Records to Characterize Patients with Uncontrolled Hypertension in Two Large Health System Networks**

**Table of Contents**

| **Content** | **First Page** |
| --- | --- |
| **Supplemental Figure S1.** Flow diagram illustrating the study process | 3 |
| **Supplemental Table S1.** Diagnosis codes for comorbidities | 4 |
| **Supplemental Table S2.** Drug ingredients in each antihypertensive drug class | 5 |
| **Supplemental Table S3.** Diagnosis codes for acute clinical outcomes | 6 |
| **Supplemental Table S4.** Flowchart to show selection of study population | 7 |
| **Supplemental Table S5.** Overlap of study population identified by approach 1 and approach 2 | 8 |
| **Supplemental Table S6.** Baseline characteristics of patients with hypertension at the index encounter (sensitivity analysis using approach 2) | 9 |
| **Supplemental Table S7.** Antihypertensive medication classes prescribed for patients with hypertension in the year prior to the index date (sensitivity analysis using approach 2) | 11 |
| **Supplemental Table S8.** Number of antihypertensive medication classes prescribed on the index date among patients with hypertension, according to age and sex (sensitivity analysis using approach 2) | 12 |
| **Supplemental Table S9.** Top three prescribed antihypertensive medication classes on the index date among treated patients with hypertension (sensitivity analysis using approach 2) | 13 |
| **Supplemental Table S10.** Rates of death, non-fatal CVD events, and healthcare utilization, among patients with uncontrolled and controlled hypertension at two health systems at 3, 6, 12, 24 months after the index date (sensitivity analysis using approach 2) | 14 |
| **Supplemental Table S11.** Baseline characteristics of patients with hypertension at the index encounter (sensitivity analysis using more than 50% of systolic blood pressure measurements below 130 mmHg and diastolic blood pressure measurements below 80 mmHg to define controlled hypertension) | 15 |
| **Supplemental Table S12.** Antihypertensive medication classes prescribed for patients with hypertension in the year prior to the index date (sensitivity analysis using more than 50% of systolic blood pressure measurements below 130 mmHg and diastolic blood pressure measurements below 80 mmHg to define controlled hypertension) | 17 |
| **Supplemental Table S13.** Number of antihypertensive medication classes prescribed on the index date among patients with hypertension, according to age and sex (sensitivity analysis using more than 50% of systolic blood pressure measurements below 130 mmHg and diastolic blood pressure measurements below 80 mmHg to define controlled hypertension) | 18 |
| **Supplemental Table S14.** Top three prescribed antihypertensive medication classes on the index date among treated patients with hypertension (sensitivity analysis using more than 50% of systolic blood pressure measurements below 130 mmHg and diastolic blood pressure measurements below 80 mmHg to define controlled hypertension) | 19 |
| **Supplemental Table S15.** Rates of death, non-fatal CVD events, and healthcare utilization, among patients with uncontrolled and controlled hypertension at two health systems at 3, 6, 12, 24 months after the index date (sensitivity analysis using more than 50% of systolic blood pressure measurements below 130 mmHg and diastolic blood pressure measurements below 80 mmHg to define controlled hypertension) | 20 |
| **Supplemental Table S16.** Baseline characteristics of patients with hypertension at the index encounter (sensitivity analysis using more than 75% of systolic blood pressure measurements below 140 mmHg and diastolic blood pressure measurements below 90 mmHg to define controlled hypertension) | 21 |

**Supplemental Figure S1.** Flow diagram illustrating the study process

**
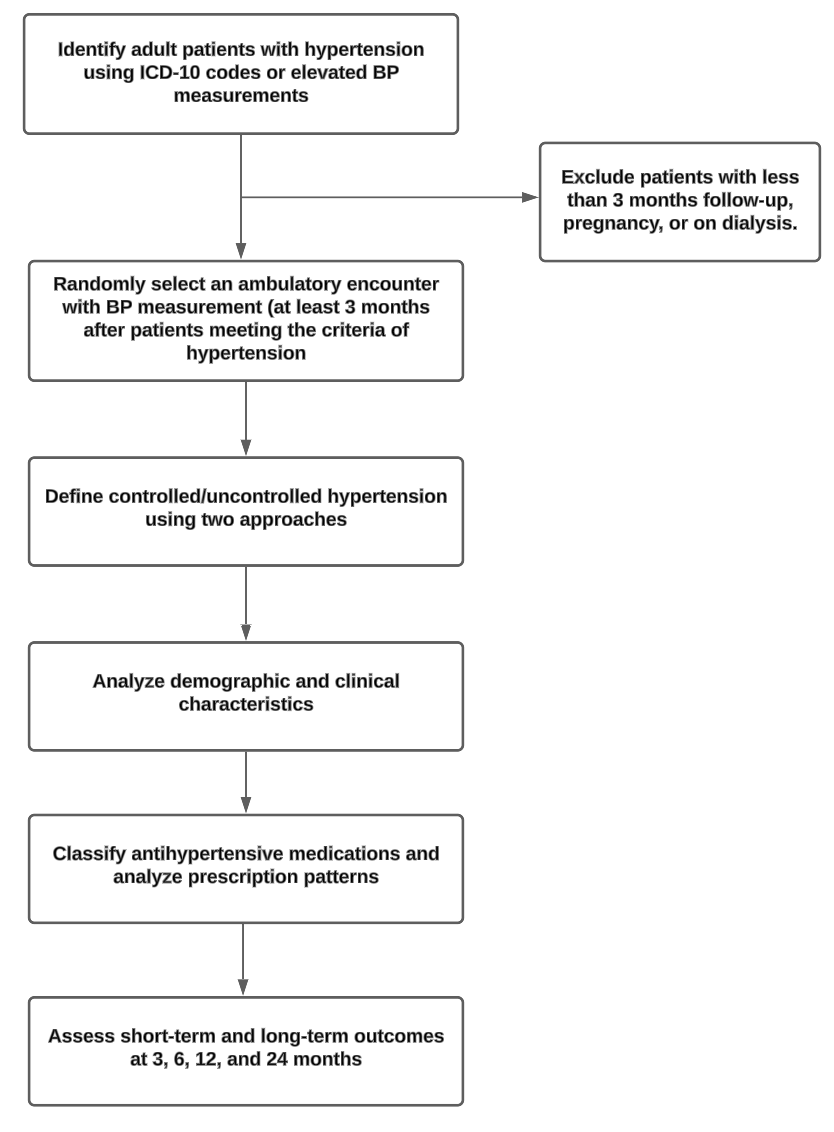
**

**Supplemental Table S1.** Diagnosis codes for comorbidities

| **Condition** | **ICD-10-CM** |  |
| --- | --- | --- |
| Heart failure | I09.9, I11.0, I13.0, I13.2, I25.5, I42.0, I42.5, I42.6, I42.7, I42.8, I42.9, I43, I50 |  |
| Diabetes mellitus | E10, E11, E12, E13, E14, E10.69, E11.69 |  |
| Dyslipidemia | E78.5, E78.2, E78.4 |  |
| Acute myocardial infarction | I21, I22 |  |
| Coronary artery disease | I25.1 |  |
| Cerebrovascular disease | I67.89, I67.9, I67.858, I69 |  |
| Atrial fibrillation/Atrial Flutter | I48 |  |
| Chronic kidney disease | N18, I12, I13 |  |
| Chronic obstructive pulmonary disease | J44 |  |
| Peripheral arterial disease | I73 |  |
| Angina | I20, I20.0, I20.1, I20.8, I20.9, I23.7 |  |
| Hemorrhagic stroke | I60, I61, I69.0, I69.1 |  |
| Ischemic stroke | I63, I69.3 |  |
| Depression | F32, F32.3, F32.8, F32.89, F32.9, F33, F33.3, F33.4 |  |
| Dementia | F01, F01.5, F02.8, F03, F03.9 |  |
| Hypertensive retinopathy | H35.03 |  |
| Substance use disorder | F10, F11, F12, F13, F14, F15, F16, F17, F18, F19 |  |

**Supplemental Table S2.** Drug ingredients in each antihypertensive drug class

| **ACEI** | **ARB** | **Beta-blocker** | **CCB** | **Thiazide or thiazide-like diuretic** | **Other antihypertensive drug ingredients** | |
| --- | --- | --- | --- | --- | --- | --- |
| Benazepril | Azilsartan | Atenolol | Amlodipine | Chlorthalidone | aliskiren | mecamylamine |
| Captopril | Candesartan | Betaxolol | Diltiazem | Hydrochlorothiazide | amiloride | methyldopa |
| Enalapril | Eprosartan | Bisoprolol | Felodipine | Indapamide | bumetanide | minoxidil |
| Fosinopril | Irbesartan | Carvedilol | Isradipine | Metolazone | clonidine | phenoxybenzamine |
| Lisinopril | Losartan | Labetalol | Nicardipine |  | doxazosin mesylate | prazosin |
| Moexipril | Olmesartan | Metoprolol | Nifedipine |  | eplerenone | reserpine |
| Perindopril | Telmisartan | Nebivolol | Nisoldipine |  | ethacrynic acid | sacubitril |
| Quinapril | Valsartan | Penbutolol | Verapamil |  | furosemide | spironolact |
| Ramipril |  | Pindolol |  |  | guanabenz acetate | spironolactone |
| Trandolapril |  | Propranolol |  |  | guanfacine | terazosin |
|  |  |  |  |  | hydralazine | torsemide |
|  |  |  |  |  | isosorbide | triamterene |

* ACEI: Angiotensin-converting enzyme inhibitor; ARB: Angiotensin receptor blocker; CCB: Calcium channel blocker.

** These drug ingredients pertaining to what was in the combination medications.

**Supplemental Table S3.** Diagnosis codes for acute clinical outcomes

| **Condition** | **ICD-10-CM** |
| --- | --- |
| Death | R99 |
| AMI | I21.0x, I21.1x, I21.2x, I21.3, I21.4, I21.9 |
| Heart failure | I50.21, I50.23, I50.31, I50.33, I50.41, I50.43, I50.811, I50.813 |
| Atrial fibrillation/Atrial Flutter | I48.0, I48.3, I48.4 |
| Aortic dissection | I71.0x, I71.1, I71.3, I71.5, I71.8 |
| Renal disease | N17.0, N17.1, N17.2, N17.8, N17.9 |
| Hemorrhagic stroke | I60.x, I61.x |
| Ischemic stroke | I63.x |
| Hypertensive urgency, Hypertensive  emergency, and Hypertensive crisis | I16.0, I16.1, I16.9 |

**Supplemental Table S4.** Flowchart to show selection of study population

| **Description** | | | **YNHHS** | **OneFlorida** |
| --- | --- | --- | --- | --- |
| **Hypertension Diagnosis** | | |  |  |
| Hypertension diagnosis at an ambulatory visit  (Oct 1, 2015 to Dec 31, 2018) | | | 346,994 | 224,534 |
| Excluded - evidence of pregnancy  (Oct 1, 2015 - Dec 31, 2018) | | | 3,462 | 1,908 |
| Excluded - dialysis  (Oct 1, 2015 - Dec 31, 2018) | | | 6,017 | 5,597 |
| Excluded - no ambulatory blood pressure measurement 3 months after identification* event and from  Oct 1, 2016 to Dec 31, 2018 | | | 112,141 | 64,489 |
|  | Excluded - met eligibility criteria and had an earlier identification^2^ date for elevated blood pressure cohort |  | 14,955 | 12,210 |
| Hypertension diagnosis cohort | | | 210,419 | 140,330 |
| **Two Elevated Ambulatory Blood Pressures** | | |  |  |
| Elevated blood pressure at two ambulatory visits  (Oct 1, 2015 to Dec 31, 2018) | | | 167,693 | 135,230 |
| Excluded - evidence of pregnancy  (Oct 1, 2015 - Dec 31, 2018) | | | 3,168 | 1,301 |
| Excluded – dialysis  (Oct 1, 2015 - Dec 31, 2018) | | | 3,485 | 2,822 |
| Excluded - no ambulatory blood pressure measurement 3 months after identification* event and from  Oct 1, 2016 to Dec 31, 2018 | | | 33,358 | 36,930 |
| Excluded - met eligibility criteria and had an earlier identification^2^ date for hypertension diagnosis cohort | | | 79,637 | 44,309 |
| Excluded - placed in hypertension diagnosis cohort if hypertension diagnosis and elevated blood pressure identification dates are the same | | | 5,257 | 7,371 |
| Elevated blood pressure cohort | | | 42,788 | 42,497 |
| **Total Patients Included in the Analysis (combining patients with hypertension diagnosis and two elevated blood pressure)** | | | 253,207 | 182,827 |

*For the hypertension diagnosis cohort, the identification date is the date of the first hypertension diagnosis associated with an ambulatory visit occurring between October 1, 2015 and December 31, 2018 while the patient is 18 years or older. For the elevated blood pressure cohort, the identification date is the date of the second elevated blood pressure measurement associated with an ambulatory visit that occur on different days and within a 6-month period between October 1, 2015 and December 31, 2018.

**Supplemental Table S5.** Overlap of study population identified by approach 1 and approach 2

| **Comparison** | **Study site** | |
| --- | --- | --- |
|  | **YNHHS**  **(N=253,207)** | **OneFlorida (N=182,827)** |
| **Overlap, n (%)** | 218,790 (86.4) | 158,688 (86.8) |

Note: Overlap in populations between approaches is defined as the patients who have the same hypertensive status (either controlled or uncontrolled) in both compared approaches. In approach 1, hypertensive patients are considered to have uncontrolled hypertension if 50% or more of their blood pressure measurements were elevated among the measured blood pressures on visits from the identification date up to and including the index date. In approach 2, hypertensive patients are considered to have uncontrolled hypertension if at the index visit their blood pressure measurement was elevated. **Supplemental Table S6.** Baseline characteristics of patients with hypertension at the index encounter (sensitivity analysis using approach 2)

| **Characteristics** | **YNHHS** | | | **OneFlorida** | | |
| --- | --- | --- | --- | --- | --- | --- |
|  | **All patients with hypertension**  **N=253,207** | **Patients with uncontrolled hypertension**  **N=77,319** | **Patients with controlled hypertension**  **N=175,888** | **All patients with hypertension**  **N=182,827** | **Patients with uncontrolled hypertension**  **N=73,072** | **Patients with controlled hypertension**  **N=109,755** |
| **Age, yrs., mean (SD)** | 65.0 (14.6) | 65.0 (14.6) | 65.1 (14.6) | 61.0 (14.7) | 61.2 (14.6) | 60.8 (14.7) |
| **Age group, N (%)** |  |  |  |  |  |  |
| 18-44 years | 21,380 (8.4) | 6,572 (8.5) | 14,808 (8.4) | 24,340 (13.3) | 9,556 (13.1) | 14,784 (13.5) |
| 45-64 years | 97,410 (38.5) | 29,920 (38.7) | 67,490 (38.4) | 82,474 (45.1) | 32,977 (45.1) | 49,497 (45.1) |
| >=65 years | 134,417 (53.1) | 40,827 (52.8) | 93,590 (53.2) | 76,013 (41.6) | 30,539 (41.8) | 45,474 (41.4) |
| **Sex, N (%)** |  |  |  |  |  |  |
| Female | 132,176 (52.2) | 39,815 (51.5) | 92,361 (52.5) | 101,006 (55.2) | 40,695 (55.7) | 60,311 (55.0) |
| Male | 121,030 (47.8) | 37,504 (48.5) | 83,526 (47.5) | 81,821 (44.8) | 32,377 (44.3) | 49,444 (45.0) |
| Other/Unknown | 1 (0.0) | 0 (0.0) | 1 (0.0) | 0 (0.0) | 0 (0.0) | 0 (0.0) |
| **Race, N (%)** |  |  |  |  |  |  |
| Black | 31,847 (12.6) | 11,669 (15.1) | 20,178 (11.5) | 46,068 (25.2) | 20,779 (28.4) | 25,289 (23.0) |
| White | 193,058 (76.2) | 57,217 (74.0) | 135,841 (77.2) | 87,118 (47.7) | 32,877 (45.0) | 54,241 (49.4) |
| Others | 24,397 (9.6) | 7,275 (9.4) | 17,122 (9.7) | 46,337 (25.3) | 18,111 (24.8) | 28,226 (25.7) |
| Unknown | 3,905 (1.5) | 1,158 (1.5) | 2,747 (1.6) | 3,304 (1.8) | 1,305 (1.8) | 1,999 (1.8) |
| **Ethnicity, N (%)** |  |  |  |  |  |  |
| Hispanic | 22,680 (9.0) | 6,781 (8.8) | 15,899 (9.0) | 28,201 (15.4) | 10,951 (15.0) | 17,250 (15.7) |
| Non-Hispanic | 222,494 (87.9) | 68,220 (88.2) | 154,274 (87.7) | 150,092 (82.1) | 60,293 (82.5) | 89,799 (81.8) |
| Other/Unknown | 8,033 (3.2) | 2,318 (3.0) | 5,715 (3.2) | 4,534 (2.5) | 1,828 (2.5) | 2,706 (2.5) |
| **Insurance type, N (%)** |  |  |  |  |  |  |
| Public (Medicare or Medicaid) | 152,866 (60.4) | 46,767 (60.5) | 106,099 (60.3) | 92,077 (50.4) | 37,311 (51.1) | 54,766 (49.9) |
| Private | 92,613 (36.6) | 28,032 (36.3) | 64,581 (36.7) | 72,434 (39.6) | 28,558 (39.1) | 43,876 (40.0) |
| Military | 1,191 (0.5) | 379 (0.5) | 812 (0.5) | 547 (0.3) | 192 (0.3) | 355 (0.3) |
| None | 3,231 (1.3) | 1,184 (1.5) | 2,047 (1.2) | 4,876 (2.7) | 2,350 (3.2) | 2,526 (2.3) |
| Others/Unknown | 3,306 (1.3) | 957 (1.2) | 2,349 (1.3) | 12,893 (7.1) | 4,661 (6.4) | 8,232 (7.5) |
| **Preferred language, N (%)** |  |  |  |  |  |  |
| English | 238,291 (94.1) | 72,654 (94.0) | 165,637 (94.2) | 163,694 (89.5) | 65,369 (89.5) | 98,325 (89.6) |
| Spanish | 9,448 (3.7) | 2,923 (3.8) | 6,525 (3.7) | 16,402 (9.0) | 6,545 (9.0) | 9,857 (9.0) |
| Others | 4,447 (1.8) | 1,430 (1.8) | 3,017 (1.7) | 2,513 (1.4) | 1,056 (1.4) | 1,457 (1.3) |
| Unknown | 1,021 (0.4) | 312 (0.4) | 709 (0.4) | 218 (0.1) | 102 (0.1) | 116 (0.1) |
| **BMI category, N (%)** |  |  |  |  |  |  |
| ≥ 30 kg/m2 | 113,301 (44.7) | 36,275 (46.9) | 77,026 (43.8) | 85,937 (47.0) | 35,617 (48.7) | 50,320 (45.8) |
| 25-<30 kg/m2 | 83,316 (32.9) | 24,878 (32.2) | 58,438 (33.2) | 55,261 (30.2) | 21,499 (29.4) | 33,762 (30.8) |
| < 25 kg/m2 | 52,487 (20.7) | 14,668 (19.0) | 37,819 (21.5) | 35,761 (19.6) | 13,450 (18.4) | 22,311 (20.3) |
| Unknown | 4,103 (1.6) | 1,498 (1.9) | 2,605 (1.5) | 5,868 (3.2) | 2,506 (3.4) | 3,362 (3.1) |
| **Smoking status, N (%)** |  |  |  |  |  |  |
| Current smoker | 8,649 (3.4) | 2,740 (3.5) | 5,909 (3.4) | 19,605 (10.7) | 8,205 (11.2) | 11,400 (10.4) |
| Former smoker | 42,557 (16.8) | 12,618 (16.3) | 29,939 (17.0) | 38,659 (21.1) | 15,050 (20.6) | 23,609 (21.5) |
| Never smoker | 8,125 (3.2) | 2,230 (2.9) | 77,026 (43.8) | 66,811 (36.5) | 27,140 (37.1) | 39,671 (36.1) |
| Unknown | 193,876 (76.6) | 59,731 (77.3) | 58,438 (33.2) | 57,644 (31.5) | 22,637 (31.0) | 35,007 (31.9) |
| **Comorbidities, N (%)** |  |  |  |  |  |  |
| Heart failure | 23,406 (9.2) | 5,715 (7.4) | 17,691 (10.1) | 17,356 (9.5) | 5,886 (8.1) | 11,470 (10.5) |
| Diabetes mellitus | 60,627 (23.9) | 18,110 (23.4) | 42,517 (24.2) | 54,696 (29.9) | 21,424 (29.3) | 33,272 (30.3) |
| Dyslipidemia | 112,082 (44.3) | 31,919 (41.3) | 80,163 (45.6) | 85,650 (46.8) | 31,661 (43.3) | 53,989 (49.2) |
| Acute myocardial infarction | 5,579 (2.2) | 1,354 (1.8) | 4,225 (2.4) | 2,834 (1.6) | 947 (1.3) | 1,887 (1.7) |
| Coronary artery disease | 43,469 (17.2) | 11,413 (14.8) | 32,056 (18.2) | 27,162 (14.9) | 9,254 (12.7) | 17,908 (16.3) |
| Cerebrovascular disease | 3,759 (1.5) | 1,119 (1.4) | 2,640 (1.5) | 3,845 (2.1) | 1,533 (2.1) | 2,312 (2.1) |
| Atrial fibrillation/Atrial flutter | 30,781 (12.2) | 7,571 (9.8) | 23,210 (13.2) | 14,951 (8.2) | 4,763 (6.5) | 10,188 (9.3) |
| Chronic kidney disease | 20,684 (8.2) | 6,453 (8.3) | 14,231 (8.1) | 20,326 (11.1) | 7,855 (10.7) | 12,471 (11.4) |
| Chronic obstructive pulmonary disease | 18,566 (7.3) | 4,854 (6.3) | 13,712 (7.8) | 14,677 (8.0) | 5,282 (7.2) | 9,395 (8.6) |
| Peripheral arterial disease | 11,272 (4.5) | 3,394 (4.4) | 7,878 (4.5) | 9,765 (5.3) | 3,791 (5.2) | 5,974 (5.4) |
| Angina | 4,074 (1.6) | 1,025 (1.3) | 3,049 (1.7) | 7,309 (4.0) | 2,588 (3.5) | 4,721 (4.3) |
| Hemorrhagic stroke | 1,421 (0.6) | 426 (0.6) | 995 (0.6) | 791 (0.4) | 299 (0.4) | 492 (0.4) |
| Ischemic stroke | 7,989 (3.2) | 2,434 (3.1) | 5,555 (3.2) | 6,172 (3.4) | 2,444 (3.3) | 3,728 (3.4) |
| Depression | 29,166 (11.5) | 7,809 (10.1) | 21,357 (12.1) | 23,189 (12.7) | 8,308 (11.4) | 14,881 (13.6) |
| Dementia | 7,713 (3.0) | 2,112 (2.7) | 5,601 (3.2) | 3,649 (2.0) | 1,351 (1.8) | 2,298 (2.1) |
| Hypertensive retinopathy | 487 (0.2) | 187 (0.2) | 300 (0.2) | 3,242 (1.8) | 1,476 (2.0) | 1,766 (1.6) |
| Substance use disorder | 29,933 (11.8) | 9,403 (12.2) | 20,530 (11.7) | 25,697 (14.1) | 10,431 (14.3) | 15,266 (13.9) |

**Supplemental Table S7.** Antihypertensive medication classes prescribed for patients with hypertension in the year prior to the index date (sensitivity analysis using approach 2)

| **Medication class** | **YNHHS** | | | **OneFlorida** | | |
| --- | --- | --- | --- | --- | --- | --- |
|  | **All patients with hypertension**  **N=253,207** | **Patients with uncontrolled hypertension**  **N=82,216** | **Patients with controlled hypertension**  **N=170,991** | **All patients with hypertension**  **N=182,827** | **Patients with uncontrolled hypertension**  **N=79,935** | **Patients with controlled hypertension**  **N=102,892** |
| Angiotensin-converting enzyme inhibitor (ACEI) | 27,803 (11) | 8,566 (11.1) | 19,237 (10.9) | 26,677 (14.6) | 11,574 (15.8) | 15,103 (13.8) |
| Angiotensin receptor blocker (ARB) | 23,439 (9.3) | 7,799 (10.1) | 15,640 (8.9) | 15,950 (8.7) | 7,446 (10.2) | 8,504 (7.7) |
| ACEI or ARB | 50,246 (19.8) | 16,027 (20.7) | 34,219 (19.5) | 41,528 (22.7) | 18,433 (25.2) | 23,095 (21) |
| Calcium channel blocker (CCB) | 29,293 (11.6) | 10,348 (13.4) | 18,945 (10.8) | 23,587 (12.9) | 11,633 (15.9) | 11,954 (10.9) |
| Beta-blocker | 38,757 (15.3) | 11,370 (14.7) | 27,387 (15.6) | 21,901 (12) | 9,434 (12.9) | 12,467 (11.4) |
| Thiazide or thiazide-like diuretic | 23,552 (9.3) | 7,921 (10.2) | 15,631 (8.9) | 22,409 (12.3) | 10,251 (14) | 12,158 (11.1) |
| Other antihypertensive drug classes | 20,562 (8.1) | 5,706 (7.4) | 14,856 (8.4) | 13,442 (7.4) | 5,762 (7.9) | 7,680 (7) |
| Combination antihypertensive drug | 13,530 (5.3) | 4,112 (5.3) | 9,418 (5.4) | 9,722 (5.3) | 4,189 (5.7) | 5,533 (5) |
| None | 157,169 (62.1) | 47,824 (61.9) | 109,345 (62.2) | 113,336 (62) | 42,927 (58.7) | 70,409 (64.2) |

**Supplemental Table S8.** Number of antihypertensive medication classes prescribed on the index date among patients with hypertension, according to age and sex (sensitivity analysis using approach 2)

1. YNHHS

| **Number of medication classes** | **Patients with uncontrolled Hypertension** | | | | | | **Patients with controlled Hypertension** | | | | | |
| --- | --- | --- | --- | --- | --- | --- | --- | --- | --- | --- | --- | --- |
|  | **Men** | | | **Women** | | | **Men** | | | **Women** | | |
|  | **18-44 years** | **45-64 years** | **65+ years** | **18-44 years** | **45-64 years** | **65+ years** | **18-44 years** | **45-64 years** | **65+ years** | **18-44 years** | **45-64 years** | **65+ years** |
| 0 | 2,362 (62.3) | 9,027 (56.6) | 10,733  (60.5) | 1,813 (65.2) | 8,563 (61.3) | 13,697  (59.4) | 5,231 (71,6) | 20,176  (61,1) | 26,241  (60.8) | 5,653 (75.3) | 23,031  (66.9) | 31,565  (62.6) |
| 1 | 811 (21.4) | 3,599 (22.5) | 3,790 (21.3) | 608 (21.9) | 2,938 (21.0) | 4,906 (21.3) | 1,273 (17.4) | 7,200 (21.8) | 9,541 (22.1) | 1,247 (16.6) | 6,636 (19.3) | 10,547  (20.9) |
| 2 | 425 (11.2) | 2,152 (13.5) | 2,037 (11.5) | 251 (9.0) | 1,636 (11.7) | 2,907 (12.6) | 572 (7.8) | 3,939 (11.9) | 4,888 (11.3) | 444 (5.9) | 3,449 (10.0) | 5578 (11.1) |
| >=3 | 192 (5.1) | 1,184 (7.4) | 1,192 (6.7) | 110 (4.0) | 821 (5.9) | 1,565 (6.8) | 229 (3.1) | 1,729 (5.2) | 2,507 (5.8) | 159 (2.1) | 1,329 (3.9) | 2,723 (5.4) |

1. OneFlorida

| **Number of medication classes** | **Patients with uncontrolled Hypertension** | | | | | | **Patients with controlled Hypertension** | | | | | |
| --- | --- | --- | --- | --- | --- | --- | --- | --- | --- | --- | --- | --- |
|  | **Men** | | | **Women** | | | **Men** | | | **Women** | | |
|  | **18-44 years** | **45-64 years** | **65+ years** | **18-44 years** | **45-64 years** | **65+ years** | **18-44 years** | **45-64 years** | **65+ years** | **18-44 years** | **45-64 years** | **65+ years** |
| 0 | 3,085 (68.0) | 9,793 (65.2) | 9,586 (74.8) | 3,338 (66.5) | 11,269  (62.8) | 12,473  (70.4) | 4,952 (77.4) | 15,427  (71.4) | 16,865  (78.6) | 6,402 (76.3) | 19,633  (70.4) | 18,207  (75.8) |
| 1 | 907 (20.0) | 2,748 (18.3) | 1,837 (14.3) | 967 (19.3) | 3,387 (18.9) | 2,769 (15.6) | 945 (14.8) | 3,465 (16) | 2,649 (12.3) | 1,309 (15.6) | 4,545 (16.3) | 3,222 (13.4) |
| 2 | 364 (8.0) | 1,560 (10.4) | 885 (6.9) | 483 (9.6) | 2,073 (11.5) | 1,538 (8.7) | 364 (5.7) | 1,864 (8.6) | 1,320 (6.2) | 491 (5.9) | 2,582 (9.3) | 1,735 (7.2) |
| >=3 | 180 (4.0) | 925 (6.2) | 507 (4.0) | 232 (4.6) | 1,222 (6.8) | 944 (5.3) | 137 (2.1) | 837 (3.9) | 619 (2.9) | 184 (2.2) | 1,144 (4.1) | 857 (3.6) |

**Supplemental Table S9.** Top three prescribed antihypertensive medication classes on the index date among treated patients with hypertension (sensitivity analysis using approach 2)

**(A) YNHHS**

|  | **All patients with hypertension**  **N=253,207** | **Patients with uncontrolled hypertension**  **N=82,216** | **Patients with controlled hypertension**  **N=170,991** |
| --- | --- | --- | --- |
| **Among adults using one medication class** |  |  |  |
| ACEI or ARB | 18,216 (34.3) | 5,904 (35.5) | 12,312 (33.8) |
| Beta blocker | 15,086 (28.4) | 4,075 (24.5) | 11,011 (30.2) |
| CCB | 10,037 (18.9) | 3,714 (22.3) | 6,323 (17.3) |
| Total | 53,097 (100) | 16,652 (100) | 36,445 (100) |
| **Among adults using two medication classes** |  |  |  |
| ACEI or ARB and Thiazide diuretic | 7,307 (25.8) | 2,450 (26) | 4,857 (25.7) |
| ACEI or ARB and Beta blocker | 3,421 (18.5) | 1,642 (17.5) | 3,597 (19.1) |
| ACEI or ARB and CCB | 3,142 (16.2) | 1,771 (18.8) | 2,802 (14.8) |
| Total | 28,278 (100) | 9,408 (100) |  |
| **Among adults using three or more medication classes** |  |  |  |
| ACEI or ARB and CCB and Thiazide diuretic | 2,321 (16.9) | 940 (18.6) | 1,381 (15.9) |
| ACEI or ARB and Beta Blocker and Thiazide diuretic | 1,913 (13.9) | 696 (13.7) | 1,217 (14) |
| ACEI or ARB and Beta Blocker and CCB | 1,722 (12.5) | 676 (13.3) | 1,046 (12.1) |
| Total | 13,740 (100) | 5,064 (100) | 8,676 (100) |

**(B) OneFlorida**

|  | **All patients with hypertension**  **N=** **182,827** | **Patients with uncontrolled hypertension**  **N=73,072** | **Patients with controlled hypertension**  **N=109,755** |
| --- | --- | --- | --- |
| **Among adults using one medication class** |  |  |  |
| ACEI or ARB | 11,638 (40.5) | 5,159 (40.9) | 6,479 (40.2) |
| CCB | 5,703 (19.8) | 2,773 (22) | 2,930 (18.2) |
| Beta Blocker | 5,406 (18.8) | 2,152 (17.1) | 3,254 (20.2) |
| Total | 28,750 (100) | 12,615 (100) | 16,135 (100) |
| **Among adults using two medication classes** |  |  |  |
| ACEI or ARB and Thiazide diuretic | 5,046 (33.1) | 2,159 (31.3) | 2,887 (34.6) |
| ACEI or ARB and CCB | 2,753 (18) | 1,396 (20.2) | 1,357 (16.2) |
| ACEI or ARB and Beta Blocker | 2,182 (14.3) | 979 (14.2) | 1,203 (14.4) |
| Total | 15,259 (100) | 6,903 (100) | 8,356 (100) |
| **Among adults using three or more medication classes** |  |  |  |
| ACEI or ARB and CCB and Thiazide diuretic | 1,629 (20.9) | 868 (21.6) | 761 (20.1) |
| ACEI or ARB and Beta Blocker and Thiazide diuretic | 985 (12.6) | 449 (11.2) | 536 (14.2) |
| ACEI or ARB and Beta Blocker and CCB | 763 (9.8) | 387 (9.7) | 376 (10) |
| Total | 7,788 (100) | 4,010 (100) | 3,778 (100) |

* ACEI: Angiotensin-converting enzyme inhibitor; ARB: Angiotensin receptor blocker; CCB: Calcium channel blocker.

**Supplemental Table 10.** Rates of death, non-fatal CVD events, and healthcare utilization, among patients with uncontrolled and controlled hypertension at two health systems at 3, 6, 12, 24 months after the index date (sensitivity analysis using approach 2)

| **Outcome (%)** | **YNHHS** | | | | **OneFlorida** | | | |
| --- | --- | --- | --- | --- | --- | --- | --- | --- |
|  | **Event rate at 3 months** | **Event rate at 6 months** | **Event rate at 12 months** | **Event rate at 24 months** | **Event rate at 3 months** | **Event rate at 6 months** | **Event rate at 12 months** | **Event rate at 24 months** |
| **All clinical outcomes*** | | | | | | | | |
| All patients | 3.3 | 5.4 | 5.4 | 8.6 | 1.9 | 2.9 | 4.3 | 6.0 |
| Uncontrolled | 2.8 | 4.8 | 4.8 | 7.9 | 1.5 | 2.3 | 3.6 | 5.3 |
| Controlled | 3.5 | 5.6 | 5.7 | 8.9 | 2.1 | 3.2 | 4.7 | 6.5 |
| **Death** | | | | | | | | |
| All patients | 1.2 | 2.2 | 2.2 | 3.7 | 1.4 | 2.3 | 3.7 | 5.7 |
| Uncontrolled | 0.8 | 1.6 | 1.6 | 2.9 | 1.1 | 1.9 | 3.1 | 4.9 |
| Controlled | 1.4 | 2.4 | 2.4 | 4.0 | 1.6 | 2.6 | 4.1 | 6.1 |
| **CVD events** | | | | |  |  |  |  |
| All patients | 2.6 | 4.1 | 4.1 | 6.5 | 2.1 | 3.3 | 5.4 | 7.8 |
| Uncontrolled | 2.3 | 3.8 | 3.9 | 6.4 | 1.9 | 3.2 | 5.2 | 7.8 |
| Controlled | 2.7 | 4.2 | 4.2 | 6.6 | 2.1 | 3.5 | 5.5 | 7.8 |
| **ED/inpatient visit for any cause** | | | | | | | | |
| All patients | 12.8 | 19.8 | 19.9 | 29.7 | 10.7 | 15.8 | 22.7 | 29.6 |
| Uncontrolled | 12.4 | 19.3 | 19.5 | 29.3 | 10.3 | 15.2 | 22.1 | 29.0 |
| Controlled | 12.9 | 19.9 | 20.1 | 29.9 | 11.0 | 16.2 | 23.2 | 30.0 |
| **Ambulatory visit for any cause** | | | | | | | | |
| All patients | 75.4 | 87.0 | 88.7 | 95.2 | 50.8 | 68.1 | 79.6 | 84.6 |
| Uncontrolled | 76.2 | 87.6 | 89.1 | 95.3 | 50.4 | 67.2 | 78.6 | 83.9 |
| Controlled | 75.0 | 86.8 | 88.6 | 95.2 | 51.1 | 68.8 | 80.3 | 85.1 |

*All clinical outcomes include the composite of death and non-fatal CVD events.

**Supplemental Table 11.** Baseline characteristics of patients with hypertension at the index encounter (sensitivity analysis using hypertension cutoffs of 130/80 mmHg and approach 1)

| **Characteristics** | **YNHHS** | | | **OneFlorida** | | |
| --- | --- | --- | --- | --- | --- | --- |
|  | **All patients with hypertension**  **N=320,248** | **Patients with uncontrolled hypertension**  **N=220,265** | **Patients with controlled hypertension**  **N=99,983** | **All patients with hypertension**  **N=231,862** | **Patients with uncontrolled hypertension**  **N=171,226** | **Patients with controlled hypertension**  **N=60,637** |
| **Age, yrs., mean (SD)** | 61.7 (16.2) | 61.8 (15.7) | 61.7 (17.2) | 58.2 (16.0) | 58.3 (15.6) | 58.1 (17.0) |
| **Age group, N (%)** |  |  |  |  |  |  |
| 18-44 years | 46,550 (14.5) | 30,206 (13.7) | 16,344 (16.3) | 44999 (19.4) | 32458 (19) | 12541 (20.7) |
| 45-64 years | 128,033 (40.0) | 91,371 (41.5) | 36,662 (36.7) | 102236 (44.1) | 77467 (45.2) | 24769 (40.8) |
| >=65 years | 145,665 (45.5) | 98,688 (44.8) | 46,977 (47.0) | 84627 (36.5) | 61300 (35.8) | 23327 (38.5) |
| **Sex, N (%)** |  |  |  |  |  |  |
| Female | 173,024 (54.0) | 116,550 (52.9) | 56,474 (56.5) | 129840 (56.0) | 95411 (55.7) | 34429 (56.8) |
| Male | 147,222 (46.0) | 103,713 (47.1) | 43,509 (43.5) | 102022 (44.0) | 75814 (44.3) | 26208 (43.2) |
| Other/Unknown | 2 (0.0) | 2 (0.0) | 0 (0.0) | 0 (0.0) | 0 (0.0) | 0 (0.0) |
| **Race, N (%)** |  |  |  |  |  |  |
| Black | 38,590 (12.1) | 29,138 (13.2) | 9,452 (9.5) | 54085 (23.3) | 43094 (25.2) | 10991 (18.1) |
| White | 243,403 (76.0) | 165,520 (75.1) | 77,883 (77.9) | 110497 (47.7) | 79785 (46.6) | 30712 (50.6) |
| Others | 33,003 (10.3) | 21,955 (10.0) | 11,048 (11.0) | 62972 (27.2) | 45176 (26.4) | 17796 (29.3) |
| Unknown | 5,252 (1.6) | 3,652 (1.7) | 1,600 (1.6) | 4308 (1.9) | 3170 (1.9) | 1138 (1.9) |
| **Ethnicity, N (%)** |  |  |  |  |  |  |
| Hispanic | 30,955 (9.7) | 20,638 (9.4) | 10,317 (10.3) | 38537 (16.6) | 27604 (16.1) | 10933 (18.0) |
| Non-Hispanic | 278,953 (87.1) | 192,390 (87.3) | 86,563 (86.6) | 187293 (80.8) | 139119 (81.2) | 48174 (79.4) |
| Other/Unknown | 10,340 (3.2) | 7,237 (3.3) | 3,103 (3.1) | 6032 (2.6) | 4502 (2.6) | 1530 (2.5) |
| **Insurance type, N (%)** |  |  |  |  |  |  |
| Public (Medicare or Medicaid) | 173,961 (54.3) | 117,570 (53.4) | 56,391 (56.4) | 107161 (46.2) | 78265 (45.7) | 28896 (47.7) |
| Private | 135,507 (42.3) | 95,409 (43.3) | 40,098 (40.1) | 102505 (44.2) | 76748 (44.8) | 25757 (42.5) |
| Military | 1,674 (0.5) | 1,092 (0.5) | 582 (0.6) | 784 (0.3) | 539 (0.3) | 245 (0.4) |
| None | 4,881 (1.5) | 3,349 (1.5) | 1,532 (1.5) | 5554 (2.4) | 4440 (2.6) | 1114 (1.8) |
| Others/Unknown | 4,225 (1.3) | 2,845 (1.3) | 1,380 (1.4) | 15858 (6.8) | 11233 (6.6) | 4625 (7.6) |
| **Preferred language, N (%)** |  |  |  |  |  |  |
| English | 299,527 (93.5) | 206,329 (93.7) | 93,198 (93.2) | 207614 (89.5) | 153842 (89.8) | 53772 (88.7) |
| Spanish | 11,805 (3.7) | 7,894 (3.6) | 3,911 (3.9) | 20914 (9) | 14987 (8.8) | 5927 (9.8) |
| Others | 5,264 (1.6) | 3,526 (1.6) | 1,738 (1.7) | 3060 (1.3) | 2189 (1.3) | 871 (1.4) |
| Unknown | 3,652 (1.1) | 2,516 (1.1) | 1,136 (1.1) | 274 (0.1) | 207 (0.1) | 67 (0.1) |
| **BMI category, N (%)** |  |  |  |  |  |  |
| ≥ 30 kg/m2 | 138,315 (43.2) | 102,174 (46.4) | 36,141 (36.1) | 103854 (44.8) | 81289 (47.5) | 22565 (37.2) |
| 25-<30 kg/m2 | 106,060 (33.1) | 72,341 (32.8) | 33,719 (33.7) | 70810 (30.5) | 51516 (30.1) | 19294 (31.8) |
| < 25 kg/m2 | 70,856 (22.1) | 42,255 (19.2) | 28,601 (28.6) | 48722 (21.0) | 32281 (18.9) | 16441 (27.1) |
| Unknown | 5,017 (1.6) | 3,495 (1.6) | 1,522 (1.5) | 8476 (3.7) | 6139 (3.6) | 2337 (3.9) |
| **Smoking status, N (%)** |  |  |  |  |  |  |
| Current smoker | 9,890 (3.1) | 6,536 (3.0) | 3,354 (3.4) | 24117 (10.4) | 18193 (10.6) | 5924 (9.8) |
| Former smoker | 47,967 (15.0) | 30,693 (13.9) | 17,274 (17.3) | 45467 (19.6) | 33310 (19.5) | 12157 (20.0) |
| Never smoker | 0 (0.0) | 0 (0.0) | 0 (0.0) | 85804 (37.0) | 64428 (37.6) | 21376 (35.3) |
| Unknown | 8,678 (2.7) | 5,291 (2.4) | 3,387 (3.4) | 76334 (32.9) | 55200 (32.2) | 21134 (34.9) |
| **Comorbidities, N (%)** |  |  |  |  |  |  |
| Heart failure | 24,207 (7.6) | 13,538 (6.1) | 10,669 (10.7) | 18579 (8.0) | 11962 (7.0) | 6617 (10.9) |
| Diabetes mellitus | 65,194 (20.4) | 44,554 (20.2) | 20,640 (20.6) | 59277 (25.6) | 43765 (25.6) | 15512 (25.6) |
| Dyslipidemia | 123,204 (38.5) | 84,877 (38.5) | 38,327 (38.3) | 94010 (40.5) | 68523 (40.0) | 25487 (42.0) |
| Acute myocardial infarction | 5,806 (1.8) | 3,383 (1.5) | 2,423 (2.4) | 3060 (1.3) | 1966 (1.1) | 1094 (1.8) |
| Coronary artery disease | 44,874 (14.0) | 27,840 (12.6) | 17,034 (17.0) | 28772 (12.4) | 19055 (11.1) | 9717 (16) |
| Cerebrovascular disease | 3,890 (1.2) | 2,540 (1.2) | 1,350 (1.4) | 4212 (1.8) | 3086 (1.8) | 1126 (1.9) |
| Atrial fibrillation/Atrial flutter | 32,288 (10.1) | 19,494 (8.9) | 12,794 (12.8) | 16102 (6.9) | 10360 (6.1) | 5742 (9.5) |
| Chronic kidney disease | 21,281 (6.6) | 13,986 (6.4) | 7,295 (7.3) | 21384 (9.2) | 15287 (8.9) | 6097 (10.1) |
| Chronic obstructive pulmonary disease | 19,900 (6.2) | 12,283 (5.6) | 7,617 (7.6) | 16180 (7.0) | 11031 (6.4) | 5149 (8.5) |
| Peripheral arterial disease | 12,048 (3.8) | 7,707 (3.5) | 4,341 (4.3) | 10482 (4.5) | 7373 (4.3) | 3109 (5.1) |
| Angina | 4,207 (1.3) | 2,593 (1.2) | 1,614 (1.6) | 7966 (3.4) | 5592 (3.3) | 2374 (3.9) |
| Hemorrhagic stroke | 1,529 (0.5) | 972 (0.4) | 557 (0.6) | 942 (0.4) | 657 (0.4) | 285 (0.5) |
| Ischemic stroke | 8,320 (2.6) | 5,539 (2.5) | 2,781 (2.8) | 6750 (2.9) | 4898 (2.9) | 1852 (3.1) |
| Depression | 36,015 (11.2) | 23,122 (10.5) | 12,893 (12.9) | 28535 (12.3) | 20247 (11.8) | 8288 (13.7) |
| Dementia | 8,058 (2.5) | 4,704 (2.1) | 3,354 (3.4) | 4041 (1.7) | 2693 (1.6) | 1348 (2.2) |
| Hypertensive retinopathy | 513 (0.2) | 379 (0.2) | 134 (0.1) | 3294 (1.4) | 2618 (1.5) | 676 (1.1) |
| Substance use disorder | 36,321 (11.3) | 24,375 (11.1) | 11,946 (11.9) | 31272 (13.5) | 22977 (13.4) | 8295 (13.7) |

**Supplemental Table 12.** Antihypertensive medication classes prescribed for patients with hypertension in the year prior to the index date (sensitivity analysis using hypertension cutoffs of 130/80 mmHg and approach 1)

| **Medication class** | **YNHHS** | | | **OneFlorida** | | |
| --- | --- | --- | --- | --- | --- | --- |
|  | **All patients with hypertension**  **N=320,248** | **Patients with uncontrolled hypertension**  **N=220,265** | **Patients with controlled hypertension**  **N=99,983** | **All patients with hypertension**  **N=231,862** | **Patients with uncontrolled hypertension**  **N=171,226** | **Patients with controlled hypertension**  **N=60,637** |
| Angiotensin-converting enzyme inhibitor (ACEI) | 27997 (8.7) | 20814 (9.4) | 7183 (7.2) | 27067 (11.7) | 21579 (12.6) | 5488 (9.1) |
| Angiotensin receptor blocker (ARB) | 23279 (7.3) | 18024 (8.2) | 5255 (5.3) | 16234 (7) | 13472 (7.9) | 2762 (4.6) |
| ACEI or ARB | 50303 (15.7) | 38101 (17.3) | 12202 (12.2) | 42170 (18.2) | 34075 (19.9) | 8095 (13.3) |
| Calcium channel blocker (CCB) | 29634 (9.3) | 23586 (10.7) | 6048 (6) | 23907 (10.3) | 20463 (12) | 3444 (5.7) |
| Beta-blocker | 40142 (12.5) | 27521 (12.5) | 12621 (12.6) | 22968 (9.9) | 17566 (10.3) | 5402 (8.9) |
| Thiazide or thiazide-like diuretic | 23489 (7.3) | 18763 (8.5) | 4726 (4.7) | 22598 (9.7) | 18933 (11.1) | 3665 (6) |
| Other antihypertensive drug classes | 21389 (6.7) | 13485 (6.1) | 7904 (7.9) | 14476 (6.2) | 10764 (6.3) | 3712 (6.1) |
| Combination antihypertensive drug | 13430 (4.2) | 10321 (4.7) | 3109 (3.1) | 9896 (4.3) | 8133 (4.7) | 1763 (2.9) |
| None | 221794 (69.3) | 148509 (67.4) | 73285 (73.3) | 159546 (68.8) | 113803 (66.5) | 45743 (75.4) |

**Supplemental Table 13.** Number of antihypertensive medication classes prescribed on the index date among patients with hypertension, according to age and sex (sensitivity analysis using hypertension cutoffs of 130/80 mmHg and approach 1)

1. YNHHS

| **Number of medication classes** | **Patients with uncontrolled Hypertension** | | | | | | **Patients with controlled Hypertension** | | | | | |
| --- | --- | --- | --- | --- | --- | --- | --- | --- | --- | --- | --- | --- |
|  | **Men** | | | **Women** | | | **Men** | | | **Women** | | |
|  | **18-44 years** | **45-64 years** | **65+ years** | **18-44 years** | **45-64 years** | **65+ years** | **18-44 years** | **45-64 years** | **65+ years** | **18-44 years** | **45-64 years** | **65+ years** |
| 0 | 12,081 (79.4) | 29,147 (64.5) | 26,891 (62.1) | 12,447 (83.0) | 32,643 (70.7) | 34,536 (62.3) | 5,445 (88.8) | 10,993 (71.6) | 14,190 (64.4) | 9,334 (91.4) | 17,245 (80.9) | 17,290 (69.3) |
| 1 | 1,909 (12.5) | 8,814 (19.5) | 9,040 (20.9) | 1,717 (11.5) | 7,702 (16.7) | 11,264 (20.3) | 470 (7.7) | 2,555 (16.6) | 4,570 (20.7) | 688 (6.7) | 2,533 (11.9) | 4,450 (17.8) |
| 2 | 860 (5.7) | 4,880 (10.8) | 4,733 (10.9) | 602 (4.0) | 4,064 (8.8) | 6,251 (11.3) | 150 (2.4) | 1,252 (8.2) | 2,215 (10.1) | 140 (1.4) | 1,144 (5.4) | 2,190 (8.8) |
| >=3 | 364 (2.4) | 2,365 (5.2) | 2,629 (6.1) | 226 (1.5) | 1,754 (3.8) | 3,344 (6.0) | 70 (1.1) | 549 (3.6) | 1,050 (4.8) | 47 (0.5) | 391 (1.8) | 1,022 (4.1) |

1. OneFlorida

| **Number of medication classes** | **Patients with uncontrolled Hypertension** | | | | | | **Patients with controlled Hypertension** | | | | | |
| --- | --- | --- | --- | --- | --- | --- | --- | --- | --- | --- | --- | --- |
|  | **Men** | | | **Women** | | | **Men** | | | **Women** | | |
|  | **18-44 years** | **45-64 years** | **65+ years** | **18-44 years** | **45-64 years** | **65+ years** | **18-44 years** | **45-64 years** | **65+ years** | **18-44 years** | **45-64 years** | **65+ years** |
| 0 | 11,909 | 81.7 | 24,915 | 72.1 | 20,719 | 77.7 | 4,252 | 91.0 | 7,929 | 79.6 | 9,452 | 81.7 |
| 1 | 1,755 | 12.0 | 5,196 | 15.0 | 3,384 | 12.7 | 307 | 6.6 | 1,242 | 12.5 | 1,265 | 10.9 |
| 2 | 635 | 4.4 | 2,882 | 8.3 | 1,681 | 6.3 | 93 | 2.0 | 561 | 5.6 | 611 | 5.3 |
| >=3 | 282 | 1.9 | 1,570 | 4.5 | 886 | 3.3 | 23 | 0.5 | 228 | 2.3 | 245 | 2.1 |

**Supplemental Table 14.** Top three prescribed antihypertensive medication classes on the index date among treated patients with hypertension (sensitivity analysis using hypertension cutoffs of 130/80 mmHg and approach 1)

**(A) YNHHS**

|  | **All patients with hypertension** | **Patients with uncontrolled hypertension** | **Patients with controlled hypertension** |
| --- | --- | --- | --- |
| **Among adults using one medication class** |  |  |  |
| ACEI or ARB | 18,442 (33.1) | 14,115 (34.9) | 4,327 (28.3) |
| Beta blocker | 16,407 (29.4) | 10,907 (27.0) | 5,500 (36.0) |
| CCB | 10,389 (18.6) | 2,038 (13.3) | 8,351 (20.6) |
| Total | 55,713 | 40,447 | 15,266 |
| **Among adults using two medication classes** |  |  |  |
| ACEI or ARB and Thiazide diuretic | 7,283 (25.6) | 5,799 (27.1) | 1,484 (20.9) |
| ACEI or ARB and Beta blocker | 5,356 (18.8) | 3,776 (17.7) | 1,580 (22.3) |
| ACEI or ARB and CCB | 4,574 (16.1) | 3,811 (17.8) | 763 (10.8) |
| Total | 28,481 | 21,390 | 7,091 |
| **Among adults using three or more medication classes** |  |  |  |
| ACEI or ARB and CCB and Thiazide diuretic | 2,317 (16.8) | 1,998 (18.7) | 319 (10.2) |
| ACEI or ARB and Beta Blocker and Thiazide diuretic | 1,959 (14.2) | 1,572 (14.7) | 387 (12.4) |
| ACEI or ARB and Beta Blocker and CCB | 1,701 (12.3) | 1,377 (12.9) | 324 (10.4) |
| Total | 13,811 | 10,682 | 3,129 |

**(B) OneFlorida**

|  | **All patients with hypertension** | **Patients with uncontrolled hypertension** | **Patients with controlled hypertension** |
| --- | --- | --- | --- |
| **Among adults using one medication class** |  |  |  |
| ACEI or ARB | 11,838 (39.1) | 9,513 (39.7) | 2,325 (36.7) |
| CCB | 5,987 (19.8) | 4,393 (18.3) | 1,594 (25.2) |
| Beta Blocker | 5,856 (19.3) | 5,006 (20.9) | 850 (13.4) |
| Total | 30,301 | 23,965 | 6,336 |
| **Among adults using two medication classes** |  |  |  |
| ACEI or ARB and Thiazide diuretic | 5,027 (32.8) | 4,128 (33.2) | 899 (31.1) |
| ACEI or ARB and CCB | 2,750 (17.9) | 2,381 (19.1) | 369 (12.8) |
| ACEI or ARB and Beta Blocker | 2,218 (14.5) | 1,669 (13.4) | 549 (19.0) |
| Total | 15,321 | 12,434 | 2887 |
| **Among adults using three or more medication classes** |  |  |  |
| ACEI or ARB and CCB and Thiazide diuretic | 1,675 (21.4) | 1,493 (22.2) | 182 (16.5) |
| ACEI or ARB and Beta Blocker and Thiazide diuretic | 974 (12.4) | 826 (12.3) | 148 (13.4) |
| ACEI or ARB and Beta Blocker and CCB | 782 (10.0) | 675 (10.0) | 107 (9.7) |
| Total | 7,831 | 6,727 | 1,104 |

* ACEI: Angiotensin-converting enzyme inhibitor; ARB: Angiotensin receptor blocker; CCB: Calcium channel blocker.

**Supplemental Table 15.** Rates of death, non-fatal CVD events, and healthcare utilization, among patients with uncontrolled and controlled hypertension at two health systems at 3, 6, 12, 24 months after the index date (sensitivity analysis using hypertension cutoffs of 130/80 mmHg and approach 1)

| **Outcome (%)** | **YNHHS** | | | | **OneFlorida** | | | |
| --- | --- | --- | --- | --- | --- | --- | --- | --- |
|  | **Event rate at 3 months (%)** | **Event rate at 6 months (%)** | **Event rate at 12 months (%)** | **Event rate at 24 months (%)** | **Event rate at 3 months (%)** | **Event rate at 6 months (%)** | **Event rate at 12 months (%)** | **Event rate at 24 months (%)** |
| **All clinical outcomes*** | | | | | | | | |
| All patients | 3.3 | 5.4 | 5.4 | 8.6 | 1.7 | 2.6 | 3.9 | 5.4 |
| Uncontrolled | 2.8 | 4.8 | 4.8 | 7.9 | 1.4 | 2.2 | 3.3 | 4.8 |
| Controlled | 3.5 | 5.6 | 5.7 | 8.9 | 2.5 | 3.8 | 5.4 | 7.3 |
| **Death** | | | | | | | | |
| All patients | 1.2 | 2.2 | 2.2 | 3.7 | 1.3 | 2.1 | 3.4 | 5.1 |
| Uncontrolled | 0.8 | 1.6 | 1.6 | 2.9 | 1.1 | 1.8 | 2.9 | 4.5 |
| Controlled | 1.4 | 2.4 | 2.4 | 4.0 | 1.9 | 3.1 | 4.8 | 7.0 |
| **CVD events** | | | | |  |  |  |  |
| All patients | 2.6 | 4.1 | 4.1 | 6.5 | 1.8 | 2.8 | 4.5 | 6.6 |
| Uncontrolled | 2.3 | 3.8 | 3.9 | 6.4 | 1.6 | 2.6 | 4.3 | 6.3 |
| Controlled | 2.7 | 4.2 | 4.2 | 6.6 | 2.3 | 3.5 | 5.3 | 7.5 |
| **ED/inpatient visit for any cause** | | | | | | | | |
| All patients | 12.8 | 19.8 | 19.9 | 29.7 | 10.2 | 15.0 | 21.5 | 27.9 |
| Uncontrolled | 12.4 | 19.3 | 19.5 | 29.3 | 9.6 | 14.4 | 20.9 | 27.3 |
| Controlled | 12.9 | 19.9 | 20.1 | 29.9 | 11.6 | 16.6 | 23.3 | 29.5 |
| **Ambulatory visit for any cause** | | | | | | | | |
| All patients | 75.4 | 87.0 | 88.7 | 95.2 | 49.6 | 66.9 | 78.6 | 83.7 |
| Uncontrolled | 76.2 | 87.6 | 89.1 | 95.3 | 49.4 | 66.7 | 78.6 | 83.9 |
| Controlled | 75.0 | 86.8 | 88.6 | 95.2 | 50.2 | 67.4 | 78.6 | 83.1 |

*All clinical outcomes include the composite of death and non-fatal CVD events.

**Supplemental Table 16.** Baseline characteristics of patients with hypertension at the index encounter (sensitivity analysis using more than 75% of systolic blood pressure measurements below 140 mmHg and diastolic blood pressure measurements below 90 mmHg to define controlled hypertension)

| **Characteristics** | **YNHHS** | | | **OneFlorida** | | |
| --- | --- | --- | --- | --- | --- | --- |
|  | **All patients with hypertension**  **N=253,207** | **Patients with uncontrolled hypertension**  **N=117,525** | **Patients with controlled hypertension**  **N=135,682** | **All patients with hypertension**  **N=182,827** | **Patients with uncontrolled hypertension**  **N=101,098** | **Patients with controlled hypertension**  **N=81,729** |
| **Age, yrs., mean (SD)** | 65.0 (14.6) | 65.3 (14.4) | 64.8 (14.7) | 61 (14.7) | 61.3 (14.5) | 60.6 (14.8) |
| **Age group, N (%)** |  |  |  |  |  |  |
| 18-44 years | 21,380 (8.4) | 9,500 (8.1) | 11,880 (8.8) | 24340 (13.3) | 12905 (12.8) | 11435 (14) |
| 45-64 years | 97,410 (38.5) | 44,896 (38.2) | 52,514 (38.7) | 82474 (45.1) | 45541 (45) | 36933 (45.2) |
| >=65 years | 134,417 (53.1) | 63,129 (53.7) | 71,288 (52.5) | 76013 (41.6) | 42652 (42.2) | 33361 (40.8) |
| **Sex, N (%)** |  |  |  |  |  |  |
| Female | 132,176 (52.2) | 61,002 (51.9) | 71,174 (52.5) | 101006 (55.2) | 56742 (56.1) | 44264 (54.2) |
| Male | 121,030 (47.8) | 56,523 (48.1) | 64,507 (47.5) | 81821 (44.8) | 44356 (43.9) | 37465 (45.8) |
| Other/Unknown | 1 (0.0) | 0 (0.0) | 1 (0.0) | 0 (0) | 0 (0) | 0 (0) |
| **Race, N (%)** |  |  |  |  |  |  |
| Black | 31,847 (12.6) | 17,307 (14.7) | 14,540 (10.7) | 46068 (25.2) | 28717 (28.4) | 17351 (21.2) |
| White | 193,058 (76.2) | 87,491 (74.4) | 105,567 (77.8) | 87118 (47.7) | 46350 (45.8) | 40768 (49.9) |
| Others | 24,397 (9.6) | 11,054 (9.4) | 13,343 (9.8) | 46337 (25.3) | 24341 (24.1) | 21996 (26.9) |
| Unknown | 3,905 (1.5) | 1,673 (1.4) | 2,232 (1.6) | 3304 (1.8) | 1690 (1.7) | 1614 (2) |
| **Ethnicity, N (%)** |  |  |  |  |  |  |
| Hispanic | 22,680 (9.0) | 10,354 (8.8) | 12,326 (9.1) | 28201 (15.4) | 14827 (14.7) | 13374 (16.4) |
| Non-Hispanic | 222,494 (87.9) | 103,747 (88.3) | 118,747 (87.5) | 150092 (82.1) | 83898 (83) | 66194 (81) |
| Other/Unknown | 8,033 (3.2) | 3,424 (2.9) | 4,609 (3.4) | 4534 (2.5) | 2373 (2.3) | 2161 (2.6) |
| **Insurance type, N (%)** |  |  |  |  |  |  |
| Public (Medicare or Medicaid) | 152,866 (60.4) | 71,845 (61.1) | 81,021 (59.7) | 92077 (50.4) | 52599 (52) | 39478 (48.3) |
| Private | 92,613 (36.6) | 42,050 (35.8) | 50,563 (37.3) | 72434 (39.6) | 38938 (38.5) | 33496 (41) |
| Military | 1,191 (0.5) | 530 (0.5) | 661 (0.5) | 547 (0.3) | 255 (0.3) | 292 (0.4) |
| None | 3,231 (1.3) | 1,627 (1.4) | 1,604 (1.2) | 4876 (2.7) | 3182 (3.1) | 1694 (2.1) |
| Others/Unknown | 3,306 (1.3) | 1,473 (1.3) | 1,833 (1.4) | 12893 (7.1) | 6124 (6.1) | 6769 (8.3) |
| **Preferred language, N (%)** |  |  |  |  |  |  |
| English | 238,291 (94.1) | 110,426 (94.0) | 127,865 (94.2) | 163694 (89.5) | 90762 (89.8) | 72932 (89.2) |
| Spanish | 9,448 (3.7) | 4,516 (3.8) | 4,932 (3.6) | 16402 (9) | 8807 (8.7) | 7595 (9.3) |
| Others | 4,447 (1.8) | 2,129 (1.8) | 2,318 (1.7) | 2513 (1.4) | 1402 (1.4) | 1111 (1.4) |
| Unknown | 1,021 (0.4) | 454 (0.4) | 567 (0.4) | 218 (0.1) | 127 (0.1) | 91 (0.1) |
| **BMI category, N (%)** |  |  |  |  |  |  |
| ≥ 30 kg/m2 | 113,301 (44.7) | 55,099 (46.9) | 58,202 (42.9) | 85937 (47) | 49230 (48.7) | 36707 (44.9) |
| 25-<30 kg/m2 | 83,316 (32.9) | 38,070 (32.4) | 45,246 (33.3) | 55261 (30.2) | 29682 (29.4) | 25579 (31.3) |
| < 25 kg/m2 | 52,487 (20.7) | 22,548 (19.2) | 29,939 (22.1) | 35761 (19.6) | 18844 (18.6) | 16917 (20.7) |
| Unknown | 4,103 (1.6) | 1,808 (1.5) | 2,295 (1.7) | 5868 (3.2) | 3342 (3.3) | 2526 (3.1) |
| **Smoking status, N (%)** |  |  |  |  |  |  |
| Current smoker | 8,649 (3.4) | 4,257 (3.6) | 4,392 (3.2) | 19605 (10.7) | 11470 (11.3) | 8135 (10) |
| Former smoker | 42,557 (16.8) | 19,832 (16.9) | 22,725 (16.7) | 38659 (21.1) | 21684 (21.4) | 16975 (20.8) |
| Never smoker | 8,125 (3.2) | 3,677 (3.1) | 4,448 (3.3) | 66811 (36.5) | 37853 (37.4) | 28958 (35.4) |
| Unknown | 193,876 (76.6) | 89,759 (76.4) | 104,117 (76.7) | 57644 (31.5) | 30045 (29.7) | 27599 (33.8) |
| **Comorbidities, N (%)** |  |  |  |  |  |  |
| Heart failure | 23,406 (9.2) | 9,363 (8.0) | 14,043 (10.4) | 17356 (9.5) | 8718 (8.6) | 8638 (10.6) |
| Diabetes mellitus | 60,627 (23.9) | 28,525 (24.3) | 32,102 (23.7) | 54696 (29.9) | 30772 (30.4) | 23924 (29.3) |
| Dyslipidemia | 112,082 (44.3) | 51,221 (43.6) | 60,861 (44.9) | 85650 (46.8) | 46110 (45.6) | 39540 (48.4) |
| Acute myocardial infarction | 5,579 (2.2) | 2,235 (1.9) | 3,344 (2.5) | 2834 (1.6) | 1432 (1.4) | 1402 (1.7) |
| Coronary artery disease | 43,469 (17.2) | 18,314 (15.6) | 25,155 (18.5) | 27162 (14.9) | 13509 (13.4) | 13653 (16.7) |
| Cerebrovascular disease | 3,759 (1.5) | 1,746 (1.5) | 2,013 (1.5) | 3845 (2.1) | 2214 (2.2) | 1631 (2) |
| Atrial fibrillation/Atrial flutter | 30,781 (12.2) | 12,453 (10.6) | 18,328 (13.5) | 14951 (8.2) | 7074 (7) | 7877 (9.6) |
| Chronic kidney disease | 20,684 (8.2) | 10,251 (8.7) | 10,433 (7.7) | 20326 (11.1) | 11582 (11.5) | 8744 (10.7) |
| Chronic obstructive pulmonary disease | 18,566 (7.3) | 7,932 (6.8) | 10,634 (7.8) | 14677 (8) | 7957 (7.9) | 6720 (8.2) |
| Peripheral arterial disease | 11,272 (4.5) | 5,438 (4.6) | 5,834 (4.3) | 9765 (5.3) | 5669 (5.6) | 4096 (5) |
| Angina | 4,074 (1.6) | 1,702 (1.4) | 2,372 (1.7) | 7309 (4) | 3842 (3.8) | 3467 (4.2) |
| Hemorrhagic stroke | 1,421 (0.6) | 640 (0.5) | 781 (0.6) | 791 (0.4) | 419 (0.4) | 372 (0.5) |
| Ischemic stroke | 7,989 (3.2) | 3,798 (3.2) | 4,191 (3.1) | 6172 (3.4) | 3539 (3.5) | 2633 (3.2) |
| Depression | 29,166 (11.5) | 12,787 (10.9) | 16,379 (12.1) | 23189 (12.7) | 12523 (12.4) | 10666 (13.1) |
| Dementia | 7,713 (3.0) | 3,269 (2.8) | 4,444 (3.3) | 3649 (2) | 1950 (1.9) | 1699 (2.1) |
| Hypertensive retinopathy | 487 (0.2) | 288 (0.2) | 199 (0.1) | 3242 (1.8) | 2132 (2.1) | 1110 (1.4) |
| Substance use disorder | 29,933 (11.8) | 14,433 (12.3) | 15,500 (11.4) | 25697 (14.1) | 14978 (14.8) | 10719 (13.1) |
